# Saturation Transfer MRI for Detection of Metabolic and Microstructural Impairments Underlying Neurodegeneration in Alzheimer’s Disease

**DOI:** 10.3390/brainsci12010053

**Published:** 2021-12-30

**Authors:** Anna Orzyłowska, Wendy Oakden

**Affiliations:** 1 Department of Neurosurgery and Paediatric Neurosurgery, Medical University of Lublin, Jaczewskiego 8 (SPSK 4), 20-090 Lublin, Poland; 2 Physical Sciences, Sunnybrook Research Institute, 2075 Bayview Avenue, Toronto, ON M4N 3M5, Canada; wendy.oakden@sunnybrook.ca

**Keywords:** Alzheimer’s disease, magnetization transfer, MT, chemical exchange saturation transfer, CEST

## Abstract

Alzheimer’s disease (AD) is one of the most common causes of dementia and difficult to study as the pool of subjects is highly heterogeneous. Saturation transfer (ST) magnetic resonance imaging (MRI) methods are quantitative modalities with potential for non-invasive identification and tracking of various aspects of AD pathology. In this review we cover ST-MRI studies in both humans and animal models of AD over the past 20 years. A number of magnetization transfer (MT) studies have shown promising results in human brain. Increased computing power enables more quantitative MT studies, while access to higher magnetic fields improves the specificity of chemical exchange saturation transfer (CEST) techniques. While much work remains to be done, results so far are very encouraging. MT is sensitive to patterns of AD-related pathological changes, improving differential diagnosis, and CEST is sensitive to particular pathological processes which could greatly assist in the development and monitoring of therapeutic treatments of this currently incurable disease.

## 1. Introduction

Alzheimer’s disease (AD) is the most common cause of dementia affecting one-third of the elderly population in the developed world and, together with other neurodegenerative diseases (NDDs), it is the fifth leading cause of death among USA seniors [1]. It is estimated that nearly 60 million people worldwide are afflicted with dementias and about 50–75% of them suffer from Alzheimer’s [2,3,4]. The clinical manifestation of AD is a progressive cognitive decline lacking symptoms which are core features of other types of dementia [5], resulting in a diagnosis of possible or probable AD. A diagnosis of AD can only be confirmed on autopsy [5], although a research framework for in vivo diagnosis based on biomarkers of neuropathology has been developed [6]. The underlying pathology consists of extracellular amyloid-β (Aβ) protein deposition, intraneuronal tau protein fibrillar aggregates (tau), as well as progressive neuronal loss, resulting in atrophy of cerebral tissue [7,8,9,10]. Complicating the situation, 10–30% of individuals diagnosed with probable AD do not display AD neuropathologic changes [6,11], while 30–40% of cognitively unimpaired elderly individuals do have AD-related neuropathologic changes at autopsy [6,12,13]. Further confusing the issue, an autopsy study by Schneider et al. has found that more than 50% of subjects with AD pathology have additional brain pathology (infarcts, Parkinson’s disease, Lewy body disease, etc.) [14]. All of this combined, makes AD a difficult disease to study as the pool of subjects is highly heterogeneous.

AD is incurable, although many attempts have been made over the years to invent treatments (please, see the reviews: [2,4,15]). The difficulty is primarily caused by the multiplicity of neuropathological processes involved and the uncertainty as to which of these processes are causes and which are effects of other pathologies [2]. Thus, the current goals in AD management are to delay the disease progression and increase life quality and expectancy, for which the continuous monitoring of at-risk populations and patients during the long prodromal period of AD is critical [6,16]. This goal was the motivation for this review, in which we aimed to evaluate the potential of saturation transfer (ST) magnetic resonance imaging (MRI) methods as emerging quantitative modalities for non-invasive radiological diagnosis and tracking of AD progression. We briefly review commonly used imaging methods for management of AD, and we describe the aspects of the pathology which are not covered by them. In this context we show how ST-MRI methods can be complementary to other modalities, as they are sensitive to microstructural tissue alterations (magnetization transfer MRI), formation of mobile proteins and their oligomers (amide proton transfer imaging), and to disturbances in the chemical and energetic balance of certain brain metabolites (chemical exchange saturation transfer MRI), all of which processes occur from the very early onset of AD pathology, and throughout the disease progression.

## 2. Pathophysiology of Alzheimer’s Disease

Neuronal death is the final stage of many of the complex pathological processes involved in AD progression, and this stage is clearly associated with extensive Aβ plaques and tau pathology [7,8,9,10]. There are many different hypotheses regarding the initial cause of AD, and the primary mechanisms driving neurodegeneration, all of which suggest different approaches to treatment [2,15,17,18]. The amyloid cascade hypothesis has been extensively investigated and is considered to be the main process in the pathogenesis of familial AD [2,15]. Mutations of the genes encoding amyloid precursor protein (APP), and presenillins 1 and 2 (PS1 and PS2, respectively) are involved in more than half of early onset (<61 years) familial AD [2,15]. These genes affect the formation of toxic forms of Aβ which then aggregate and form amyloid plaques [17,19]. The hypothesis is that these plaques then interact with both glial and neuronal cells, leading to neuroinflammation, hyperphosphorylation of tau, synaptic injury, vascular impairment, and contributing to mitochondrial dysfunction, intensification of oxidative stress, and increased neuronal apoptosis [2,17].

Sporadic AD, which accounts for 95% of all cases, is still not fully understood, with many different factors involved in pathogenesis, although the order in which these pathological events occur is quite variable [15].

One of the hypotheses of sporadic AD etiology is that neuroinflammation occurs long before protein accumulation, as the immune system first attempts to remove increasing amounts of Aβ (neuroprotective effect), but as the disease develops, many different stages of glial activation occur, significantly contributing to neurodegeneration [20,21]. Extensive activation of glial cells leads to gliosis (micro- and astrogliosis), in which the secretion of cytotoxic compounds by glial cells exacerbates neuroinflammation, decreases the effectiveness of Aβ removal and contributes to plaque formation. Exposure to pro-inflammatory factors also causes astroglial injury and progressive atrophy [22]. Long-term astroglial impairment contributes to neurodegeneration by disrupting the blood brain barrier, disturbing brain homeostasis, and reducing the supply of nutrients to neurons. The malfunctioning of astroglia also causes the accumulation of glutamate in extracellular space leading to excitotoxicity. All these events are already taking place in the early stages of AD [22]. As the astroglia are responsible for maintaining synaptic connections, astroglial atrophy causes synaptic loss, which is one of the leading processes resulting in long-term cognitive impairment [21,22].

This spreading pattern of synaptic density loss is similar to the progressive occurrence of such tau protein positive structures as intraneuronal neurofibrillary tangles (NFTs), neuropil threads, and neuritic plaques [10,21]. This indicates a strong relationship between tau pathology and cognitive decline [23] and constitutes the tau hypothesis of AD pathogenesis. There is evidence that AD dementia is even more closely associated with tauopathy than with amyloidosis [23,24,25]. AD tauopathy occurs mainly as a consequence of neuroinflammation caused by emerging Aβ, which intensifies the phosphorylation of tau protein, leading to the formation of NFTs within neuronal bodies [15,21]. The other possible factor contributing to tau aggregation is microglial senescence [25]. The presence of phosphorylated tau (p-Tau) tangles compromises the biochemical exchange between intra- and extracellular compartments and causes damage to the cytoskeleton which contributes to p-Tau leakage and further neurodegeneration [2,15]. According to Braak’s classification, the first signs of tau pathology occur in subcortical areas, and the locus coeruleus. Pathology then spreads to transentorhinal regions and progresses to neocortex, where the first signs of Aβ arise, while neuropathology further affects other brain areas [10]. However, recently Vogel et al. pointed out four dominant phenotypes of tau spreading patterns, among which limbic-predominant, medial temporal lobe-sparing, posterior, and lateral temporal trajectories are the most commonly seen among patients [23].

In the mitochondrial cascade hypothesis, mitochondrial inhibition, disturbed redox homeostasis, and impaired energy metabolism are implicated in sporadic AD pathogenesis. The dysfunctional mitochondria contribute to neurodegeneration by upregulating the production of reactive oxygen species (ROS), misproduction of adenosine triphosphate (ATP), disturbance in calcium ions (Ca^2+^) homeostasis, compromised mitochondrial dynamics and insufficient mitophagy [26]. All these events are observed already in early stage AD, resulting in insufficient energy storage and decrease the ability of the cell to metabolize glucose [26,27]. The altered mitochondrial bioenergetics and oxidative stress cause extensive oxidative damage to lipids, proteins and DNA (nuclear and mitochondrial), exacerbate Aβ aggregation and tau hyperphosphorylation. Mitochondrial pathology correlates with synaptic dysfunction and dendritic branch degeneration in AD [27,28,29].

Other hypotheses of AD pathogenesis include the emerging role of acetylcholine neurotransmitter deficiency, decreased microRNA levels, and acute vitamin B5 deficiency [2], but describing them is beyond the scope of this review.

All the above play concurrent roles and occur without consistent chronology, but they do contribute significantly to over-stimulation of neuronal apoptosis and result in degeneration of cerebral tissue [2]. These processes are already occurring in preclinical stage of AD [30] which can last up to 20 years from the onset of neuropathological changes [1]. Mild cognitive impairment (MCI), specifically amnestic MCI (aMCI) is considered the first symptomatic phase of these underlying neuronal changes, being a prodromal stage of AD [31,32]. The disease manifests clinically as progressive dementia, which is usually assessed by Mini-Mental State Examination (MMSE), and comprises as memory loss, difficulties with logical thinking, language problems, and behavioral changes, altogether posing significant obstacles to everyday activities [1].

In order to assist researchers in targeting the biology underlying AD rather than the symptoms of dementia, a biologically based research framework has been developed, which relies on biomarkers sensitive to the aggregated Aβ (A), aggregated tau (T), and neurodegeneration or neuronal injury (N) (AT(N) scale) [6]. In the next chapter we depict the current clinical practice for assessing these biomarkers non-invasively, and discuss the possibilities for detecting the remaining aspects of the pathology using the accessible imaging modalities.

## 3. Imaging Techniques in Current Clinical Practice

### 3.1. PET

Positron emission tomography (PET) requires intravenous injection of a radioactive tracer, which accumulates in tracer-specific locations. A PET imaging system is then used to visualize the tracer accumulation. Three different types of PET imaging are currently used in the assessment of AD: glucose metabolism using [18F]-2-fluoro-2-deoxy-D-glucose (FDG) PET (FDG-PET), and amyloid or tau deposition using amyloid-β PET (Aβ-PET) or Tau-PET [33,34,35].

In AD, glucose hypometabolism is observed in both parietal and temporal lobes areas, posterior cingulate cortex, and medial temporal lobe. Cerebellum, striatum, basal ganglia, primary visual, and sensorimotor cortices are not typically affected [36,37]. FDG-PET is very sensitive to AD, predicts progression from MCI to AD with relatively high specificity, and can distinguish between different types of dementia [33,35].

Aβ-PET allows for in vivo detection of amyloid plaques [33,34]. There are several different tracers which bind to Aβ [35], but quantification and standardization of cut-off levels for different tracers still need to be defined [33,38].

While Tau-PET shows great potential as a clinical tool, development of Tau-PET tracers lags behind tracers for Aβ, and issues with off-target binding, and regional specificity of tau are still being resolved [34].

Although PET imaging sensitivity and specificity is high, it does involve radiation exposure and should not be used too frequently [34]. Moreover, its spatial resolution is relatively poor. The other drawbacks of PET imaging in everyday practice are cost and availability [34,35].

### 3.2. SPECT

Single-photon emission computed tomography (SPECT) is an imaging modality similar to PET in that it also uses radioactive tracers to visualize abnormal protein deposits or target molecular processes [33]. SPECT uses gamma-emitting as opposed to positron-emitting radioisotopes, which typically have longer half-lives than PET tracers [39]. SPECT is less expensive than PET but at the cost of decreased contrast and spatial resolution. While PET can be used to assess hypometabolism, SPECT is commonly used to detect hypoperfusion [33]. Currently perfusion SPECT is recommended as an alternative to FDG-PET for the differential diagnosis of AD [40,41].

### 3.3. MRS

Magnetic resonance spectroscopy (MRS) is a method for evaluating metabolites’ levels in the brain non-invasively. Since changes in metabolism precede any structural changes in AD, MRS may appear to be an ideal technique for AD diagnosis. A recent review of MRS in common dementias has found decreased N-acetylaspartate (NAA) in posterior cingulate cortex, hippocampus, temporal, and parietal lobes in patients with AD [42]. Increased myo-inositol (mIns) and total choline levels, relative to total creatine (tCr), are also observed in the posterior cingulate cortex [42]. However, the utility of MRS in prediction or differential diagnosis of AD has been hampered by a number of technical issues. The signal measured by MRS is low compared to that measured with MRI, meaning that it takes considerably longer to acquire, and only some metabolites are present in large enough quantities to be measured [43]. MRS typically measures only one voxel at a time, so most research has focused only on a few select brain regions, making meta-analyses difficult [42]. Magnetic resonance spectroscopic imaging (MRSI) can solve this problem, but at the cost of reduced data quality compared with MRS [44]. Finally, there are technical issues surrounding both data acquisition [45] and analysis [46], which make it difficult to compare data from different studies. In summary, MRS is often limited in both resolution and sensitivity due to its inherently low signal. Single voxel techniques are good at quantifying metabolites, but are often not informative due to tissue heterogeneity. MRS imaging, due to its decreased signal, allows for semi-quantification of only a few metabolites (NAA, creatine, choline [47], and D-2-hydroxyglutarate [48]) with high variability (15–20%) [48], while suffering from relatively coarse image resolution and limited brain coverage. MRS allows for group comparisons, but often fails at individual diagnosis.

As the quality and interpretability of MRS are strongly related to the strength of the magnetic field [49], as higher field MRI scanners become increasingly available, MRS results in AD will likely improve.

### 3.4. Other MRI Methods

Structural imaging is used to identify brain lesions, determine patterns of atrophy, and look for signs of vascular disease, in order to rule out other causes of dementia [34,35]. Measurements of hippocampal and entorhinal cortex volumes obtained from structural MR images can be used to differentiate patients with AD [50,51].

Increased iron deposition in the amygdala and caudate nucleus, likely associated with the presence of Aβ, have been observed in AD patients using quantitative susceptibility mapping MRI techniques [52].

Direct imaging of Aβ has also been attempted with MRI. While several MR parameters are sensitive to the changes associated with amyloid [51,53] MR contrast agents have been developed which bind to Aβ plaques [51]. However, none of these agents are currently available for human use [51,53].

Functional magnetic resonance imaging (fMRI) excels at localizing brain function, and can potentially identify disease-related differences in function, however it is only a relative measure of neural activity as it detects changes in blood oxygenation [54,55]. Task-based fMRI has demonstrated increased fMRI signal in MCI subjects relative to both healthy control and AD subjects, complicating the interpretation of fMRI studies of AD [56]. Functional connectivity of the brain, assessed using resting-state fMRI, can reveal intrinsic connectivity networks [57], and demonstrates changes in connectivity in AD [58,59].

Connectivity and white matter microstructure are evaluated using diffusion tensor imaging (DTI) which is sensitive to the microscopic motion of water molecules [60,61,62,63]. Diffusivity of water is increased in the hippocampus and white matter of AD patients indicating decrease in cellularity [50].

Arterial spin labelling (ASL) is another promising MRI-based technique that measures blood flow in the brain. It does not require an exogenous contrast agent, and can produce maps of cerebral perfusion similar to SPECT but with higher resolution [64]. As perfusion and metabolism are linked, it can also offer an alternative to FDG-PET for measuring the patterns of hypometabolism associated with AD [65,66,67,68].

### 3.5. Imaging Other Aspects of AD Pathology

There are several neuropathological processes which are not covered by the above imaging modalities, specifically neuroinflammation and mitochondrial dysfunction. Given the concurrent role of these processes in AD development one goal should be also to find new non-invasive biomarkers of these processes. For example, a biomarker for neuroinflammation in the prodromal stage of AD, which would distinguish between neuroprotective and detrimental stages of inflammation could allow them to be targeted separately by novel therapies [21]. Such achievements may provide control over interactions between the immune and nervous systems in early stage AD to prevent later irreversible stages of neural degeneration [21]. Comprehensive studies of the role of mitochondrial dysfunction in AD pathogenesis are emerging to target mitochondria for preserving energy metabolism in new therapies [26]. Having an imaging tool, which does not involve radiation risk, would help to achieve this goal. It seems that saturation transfer MRI techniques have the potential to fill this gap, as they are molecular imaging methods which do not require contrast injection. By measuring signal originating from semi-solid or mobile macromolecules, proteins and metabolites they are reflecting tissue microstructure and metabolic processes. In the next sections we will describe these methods, including principles of acquisition setup and data analysis, and then we will discuss the findings in AD based on ST-MRI studies found in literature.

## 4. Magnetization Transfer (MT) MRI

### 4.1. Principles of the MT Acquisition

Magnetization transfer, MT, is a contrast mechanism that is sensitive to the concentration of macromolecular protons (mainly associated with cell membranes) and their exchange with free water protons [69,70]. Both, MT and chemical exchange saturation transfer, CEST, experiments measure the transfer of magnetization from molecular protons to the solvent water protons, an effect that becomes apparent as an MRI signal loss (i.e., saturation). This allows molecular information to be accessed with the enhanced sensitivity of MRI. The only difference between MT and CEST is the type of molecules probed: macromolecules (mostly lipids associated with cell membranes in MT) and small, mobile proteins (in CEST) [71]. They also use the same saturation transfer MRI sequences, albeit with slightly different experimental parameters. In reality, CEST measurements are always influenced by MT and therefore in more advanced CEST studies MT has been extensively used to probe white matter integrity in which it reveals myelin content [70,72,73] while CEST is popular in cancer and stroke [74,75,76,77,78].

### 4.2. MT Data Processing and Parametrization

An MT experiment uses a single off-resonance saturation radiofrequency (RF) pulse, of frequency well away from that of the water, and results in an image with decreased signal in areas with a lot of macromolecules (e.g., white matter). An identical image with no saturation can also be acquired, and used to calculate the MT ratio (MTR):

(1) $$\mathrm{MTR}=\frac{S_{0}-S_{sat}}{S_{0}}=1-\frac{S_{sat}}{S_{0}}$$

where S_0_ is the signal without saturation, and S*_sat_* is the signal with saturation. MTR images can be generated by calculating the MTR at each voxel in the image and have positive contrast in areas with an abundance of semi-solid macromolecules. In the brain, for example, an MTR image has the most signal in healthy white matter, and decreased signal in areas where loss of myelin has occurred.

In order to compare MTR values between subjects or studies, the sequence parameters must be identical. MT contrast is affected not only by the underlying tissue, but also by the frequency, power, shape, and duration of the MT saturation pulse. The specific imaging sequence used can also affect the results. It is possible to take advantage of these changes in MT contrast due to frequency and power of the saturation pulse, and acquired several different data sets in order to fit a quantitative model and attempt to quantify the underlying biological parameters.

Quantitative MT (qMT) is typically based on a two-pool model initially developed by Henkelman et al. [79]. The two pools in the model are the liquid or “free water” pool, and the macromolecular or “bound water” pool. Each pool has a spin density, M_0_, a longitudinal relaxation rate R_1_, and a transverse relaxation time T_2_. These pools are typically denoted as A and B, or f (free) and r (restricted). Finally, there is the parameter R which is the rate at which magnetization is exchanged between the two pools, which is sometimes replaced by k_f_ and k_r_ which are the forward and reverse exchange rate constants [73]. In order to fit these parameters to experimental data, a set of MT images are acquired over a range of different offsets, using at least two different saturation RF pulse powers, sometimes reported as flip angles. B_1_ and T_1_ maps are also collected in order to correct for variations in RF power across the image, and to constrain the fitting. Even with all of this data, it is still challenging to fit all 7 model parameters as some of them are tightly coupled. As a result, the parameters which are usually reported are the product of the exchange rate and the spin density of the macromolecular pool, R × M_0_^B^, the ratio of the macromolecular pool size to the water pool, F, and the T_2_ of the macromolecular pool, T_2_^B^ [71,79].

In healthy brain R × M_0_^B^ and F are highest in white matter, while T_2_^B^ is relatively uniform across the brain. A decrease in RM_0_^B^ is due either to an increased exchange rate or a decrease in molecular fraction, M_0_^B^. This might reflect a breakdown of lipid membranes, demyelination, or axonal loss. A decrease in F indicates either a decrease in macromolecular fraction, M_0_^B^ which is due to demyelination, or an increase in M_0_^A^ which may be the result of inflammation [80,81,82].

### 4.3. MT-Based Findings in Alzheimer’s Disease

To date there have been several studies using MTR to investigate AD patients, or subjects with a high risk of developing AD. These studies are listed in Table 1, along with the field strength, subjects, and brain regions analyzed.

These MT studies have been based on either fast spin echo (FSE) or spoiled gradient recalled (SPGR) sequences with a variety of different MT pre-pulses. Most studies cover the whole brain with a resolution typically 1 × 1 mm^2^ in plane and 3–5 mm through plane. Saturation parameters differ between studies and are not always provided, making comparisons of MTR values between studies difficult. Most studies report mean MTR values, while some also include histogram analyses of larger regions.

**Figure 1:**
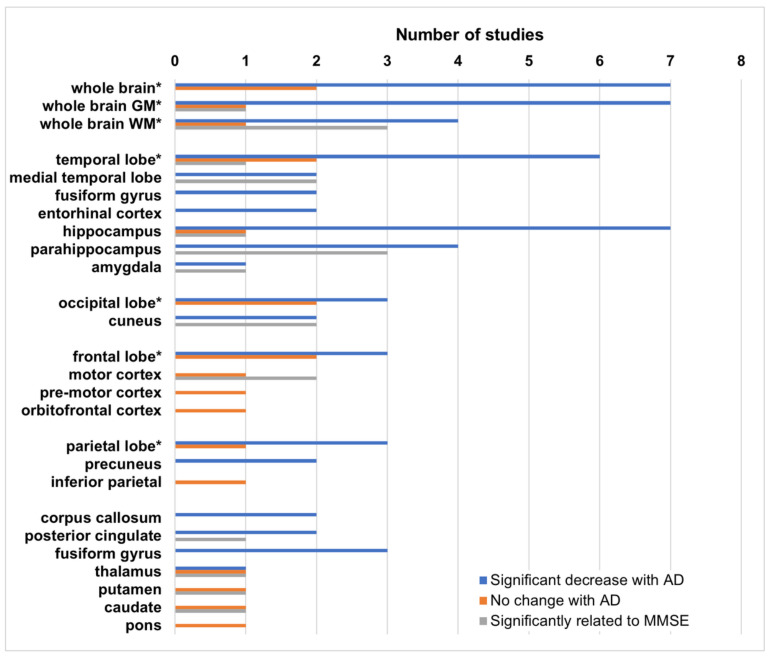
Studies report significant decreases in magnetization transfer ratio (MTR) and/or histogram peak height related to Alzheimer’s disease (AD) and mini-mental state examination (MMSE) in a variety of different brain regions, most of which are included in this figure. For the two studies which have analyzed only mild cognitive impairment (MCI) subjects, significant decreases in MTR vs. controls are also included. (* numbers include both MTR and histogram peak heights. Peak heights are more sensitive to changes, and some studies report both a significant decrease in peak height and non-significant change in MTR in the same region). GM—gray matter; WM—white matter.

The most common result in all of these studies is a significant decrease in either MTR, or histogram peak height, in AD patients relative to healthy controls in at least one brain region. When MCI subjects are included, their MTR values are lower than those of healthy controls, but higher than those of the AD patients, although these differences do not typically achieve significance. Histogram peak height is more sensitive to small changes over large regions, likely indicative of regional changes. Regional analyses using ROIs or subregions are performed primarily in areas known to be affected by AD, although some unaffected regions are included as controls in some studies. Results of these regional analyses are shown in Figure 1.

While most studies have focused on both, gray and white matter changes, some concentrate exclusively on either white or gray matter. Because MTR is, in part, related to myelin [72], changes in WM associated with demyelination are likely to be much larger than in gray matter, however MTR decreases in gray matter appear to be more sensitive to MMSE status [86,91,94,98]. In addition, several studies have found that these decreases are not necessarily symmetric, with larger decreases most often found in the left hemisphere [94,95,96,98].

Two studies also include subjects with non-AD dementia. Hanyu et al. show that MTR in the hippocampus is significantly lower with respect to vascular and other non-AD dementias [84], as well as in dementia with Lewy bodies (DLB) [89].

Ropele et al. [94] is the only group to conduct a longitudinal study. While their baseline data initially show no change in MTR in the thalamus, caudate, and putamen at baseline, paired *t*-tests at later timepoints demonstrate a significant decrease in the thalamus and caudate after 6 months, and in the putamen at 12 months, suggesting that longitudinal studies are more sensitive to MTR changes, particularly in gray matter regions.

Fornari et al. [95] have found different patterns of decreased MTR in subcortical white matter depending on whether the Alzheimer’s disease is early vs. late onset. Carmeli et al. [97] have found regions of decreased MTR specific to impairment of particular cognitive domains (e.g., decreased MTR in prefrontal white matter, and insula is specific to executive dysfunction).

In spite of the variability in disease severity, MT saturation methods, brain segmentation, and choice of regions for analysis, MTR is very consistently observed to decrease in AD subjects relative to healthy controls in particular brain regions. This suggests that saturation transfer techniques are sensitive to the changes associated with AD. Regional analyses of the brain with separate gray and white matter MTR values, while more complicated, are likely to lead to more interesting results. Another approach to increase the sensitivity and specificity of MT measurements, is to move from simple MTR acquisitions to quantitative MT.

Only five human studies of AD using quantitative MT have been found in the literature. While the results are promising, the data collection is lengthy, and analysis is complicated. Software to fit the qMT model has been implemented by each individual lab, as commercial software is not available. It is difficult to directly compare these studies as they each report slightly different parameters in different brain regions. Giulietti et al. [99] is the only group to perform a full brain analysis, all other groups use pre-selected ROIs. The results of these studies are listed in Table 2.

In spite of the variability in these analyses, all studies report changes in qMT parameters. The most consistent result is a decrease in the MT exchange parameter, R × M_0_^B^, in many regions strongly associated with AD pathology. This appears to be more sensitive than MTR as it can not only differentiate between AD and control subjects [99,100], but also differentiate MCI subjects from both AD and controls [101,102,103]. Such differentiation between NC, MCI, and AD was not possible when using MTR rather than qMT. The relationship between MMSE scores and qMT parameters is only reported by Ridha et al. [100], who found that F × T_1_^A^ (denoted by authors as f*_b_), is correlated with MMSE in the hippocampus in AD patients.

The work by Wiest et al. [102] suggests that there are changes in all of the fitted qMT parameters, that while these changes may not be statistically significant on their own, machine learning can help to identify patterns in these parameters which are indicative of AD or MCI. The increased sensitivity makes qMT a promising research tool, however the time needed to acquire the data, and the expertise required to analyze the data means that it is not ready for clinical use.

Magnetization transfer studies in animal models of AD have been performed at both 9.4 T and 7 T (Table 3), in three different mouse models with mutations causing them to overexpress human Alzheimer Aβ precursor protein (APP), and/or presenilin 1 (PS1), both of which are involved in familial AD. The Tg2576 mouse overexpresses one particular isoform of human APP, and develops Aβ plaques but not tau [104]. The APP/PS1 mouse model is based on the Tg2576 model crossed with a PS1 mutant, and exhibits both Aβ and tau pathology [105,106]. The third model is a BRI-Aβ-42 (BRI) mouse, which exhibits a later onset of Aβ deposition [107]. Finally, the Tg/SOD model is a cross between the Tg2576 and a model which overexpresses the mitochondrial antioxidant superoxide dismutase 2 (SOD), which has reduced AD pathology and improved cognition [108].

While human studies all observe decreased MTR in various brain regions, animal studies report the opposite. Increased MTR is observed in both cortex and hippocampus of Tg2576 mice as early as 6 months [108], and in APP/PS1 mice at 12 months [109] and 18 months [110]. As expected, no changes are observed in the Tg/SOD mice [108], or the BRI mice [110]. Interestingly, although an increase in cortical MTR is noticed by Praet et al. [111] at 24 months, they find decreased MTR in the hippocampus as a genotype effect of APP/PS1 which is in contrast with previous results. They also observe decreased MTR in both genu and splenium of the corpus callosum, evident as early as 2 months [111], which is correlated with Aβ plaque deposition, astro-, and microgliosis, but not with myelin basic protein. There was some confusion regarding the results from Esteras et al. [109] because MT changes were reported as the ratio of saturated to unsaturated signal rather than MTR which uses the difference between unsaturated and saturated signals as the numerator.

The increase in MTR is postulated by Bigot et al. [110] to be due to differences in the hydrophobicity of Aβ plaques [112]. Human Aβ plaques are extremely hydrophobic, which may reduce the exchange rate and decrease MTR, while the mouse Aβ plaques are much more soluble, and this decreased hydrophobicity could contribute to increased MTR. Praet et al. [111] also note that the APP/PS1 model is a very aggressive one, with changes beginning as early as 6 weeks, prior to brain maturation. As changes of myelination continue after 6 months [113], and MT is sensitive to myelin [72], MT may be a poor choice of contrast in this particular model.

## 5. Chemical Exchange Saturation Transfer (CEST)

### 5.1. CEST Principles

CEST contrast can be added to any MR imaging sequence by adding a saturation pulse identical to the saturation pulse used to generate MT contrast, but at a frequency much closer to that of water, and typically at a lower power. In CEST experiments the mechanism by which magnetization is transferred from metabolites or contrast agents to water molecules is via the chemical exchange of protons [114]. This can either happen directly, with the saturated protons being exchanged, as with amides, or indirectly as with aliphatic protons which are not exchangeable but interact with other exchangeable protons in the protein which then relay that saturation effect to water which is called the nuclear Overhauser effect (NOE). This exchange of saturated protons then reduces the number of protons contributing to the water signal when the image is acquired. The amount by which the magnetization is decreased by CEST depends on both the concentration of the protein or metabolite, and the rate at which it is exchanging with water. Other contributing factors include the amount of RF power (B_1_), the duration, and the bandwidth of the saturation pulse. These are often limited by the hardware available, and by safety considerations as very long, high powered RF pulses induce heating [71].

The CEST effect from many brain metabolites is detectable using currently available MRI equipment, in both clinical and experimental systems (Lee2016).

CEST frequency offsets are typically reported in parts per million (ppm), with water at 0 ppm rather than at 4.7 ppm as is done in MRS. The ppm scale is useful as it does not depend on magnetic field strength.

**Figure 2:**
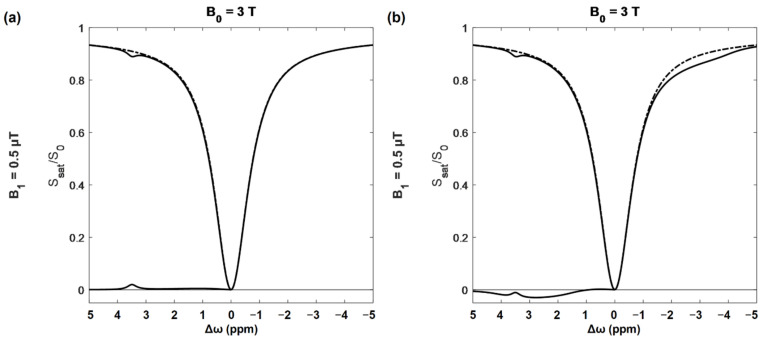
Simulated z-spectrum and MTR asymmetry (MTR_asym_). Dashed line contains contributions from water and magnetization transfer (MT). Solid line contains additional contributions from amide, glutamate, guanidine, myo-inositol (**a**,**b**), as well as from nuclear Overhauser effect (NOE) (**b**). Corresponding MTR_asym_ plots are located in the bottom left corner of both plots. Most of the CEST peaks are either too small, or too close to the water peak to be easily visible at 3T magnetic field (B_0_), but the amide peak at 3.5 ppm is quite clearly seen here. B_1_—the amplitude of saturation radiofrequency pulse; S_0_—signal without saturation; S_sat_—signal with saturation; Δω—frequency offset in reference to water resonance.

A CEST spectroscopic experiment involves acquiring a set of saturation transfer weighted MR images with one saturation power but many offsets, typically ranging between 5 and −5 ppm. For each voxel the ratio of saturated to unsaturated signal is calculated, and then plotted vs. the offset frequency to generate a z-spectrum (Figure 2).

The very large dip at the center of the z-spectrum (Figure 2) is the result of saturating at, or very near, the water frequency, and is referred to as the direct effect (DE). The width of the DE peak depends mainly on the power and bandwidth of the saturation pulse and T_1_/T_2_ ratio of the tissue [79]. At very large offsets the signal tends towards 1. Frequency offsets are plotted with positive frequencies to the right of water as is conventional in MR spectroscopy. MT contrast resulting from the presence of macromolecules cannot be eliminated and increases with saturation power. This effect is quite broad, and approximately symmetric about the water frequency [79]. MTR asymmetry (MTR_asym_) is given by:

(2) $${\mathrm{MTR}}_{\mathrm{asym}}(\Delta \omega ):\;\frac{S\left(-\Delta \omega \right)-S\left(+\Delta \omega \right)}{S_{0}}\times 100\%$$

where S (±Δω) is the saturation transfer signal at that frequency offset, and S_0_ is the unsaturated signal. Often S_0_ is replaced by a saturated signal with saturation frequency far enough from water that CEST effects are expected to be minimal (e.g., >8 ppm) [71].

MTR_asym_ spectra, where the signal to the left of water is subtracted from the right, are often used in order to eliminate MT from the CEST signal. Unfortunately, NOE contributes signal to the right of the water peak, which causes a fairly broad decrease in the z-spectrum between −1 and −5 ppm [71]. As shown in Figure 2b, these can substantially alter the MTR_asym_ spectrum, and affect the specificity of CEST results when doing asymmetry-based analysis.

### 5.2. Key Points of CEST Data Acquisition, Analysis, and Parametrization

Each offset frequency in the CEST spectrum requires a different saturation pulse and must be obtained as part of a separate image, so a highly sampled spectrum can take a long time to acquire. The simplest approach is to measure the z-spectrum, collecting enough offset images to perform a correction of B_0_ field inhomogeneities [71,74].

In order to simplify interpretation of the data, images are generated based on the CEST contrast at the offset of interest, which can be calculated in a number of different ways. For amide proton transfer (APT) imaging, the offset of interest is 3.5 ppm. The APT contrast is then calculated based on either the MTR_asym_ value at 3.5 ppm, the area under the curve (AUC) between 3.3 and 3.7 ppm, or by fitting various components of the z-spectrum and calculating the area of the peak [71,115].

A number of other types of CEST contrast can be acquired in a similar fashion. Creatine CEST (CrCEST) is based on the guanidinium peak at 2 ppm, myo-Inositol CEST (MICEST) on the peak at 0.9 ppm, glutamate CEST (gluCEST) at 3 ppm, and glucose CEST (glucoCEST) at 1.2 ppm. These different CEST images are generally named after the dominant component of the signal at the frequency of interest; however, it is important to remember that a variety of different effects contribute to the signal [116].

There are many different approaches aiming to remove or correct for unwanted signals in CEST experiment [71]. Many of these approaches rely on collecting the z-spectrum as described above and attempting to fit the data to a particular physical model. Others such as SAFARI (saturation with frequency alternating RF irradiation) [117] or VDMP (variable-delay multipulse) [118] try to remove unwanted signal more directly by modifying the pulse sequence. These approaches, while interesting, are not always easier to interpret [119].

There are two sources of CEST effect: endogenous and exogenous. Endogenous CEST probes the metabolites in the tissue, while exogenous CEST measures the effect of CEST specific contrast agents, and also can be used to increase the CEST effect from intrinsic metabolites.

### 5.3. Animal Models Used in CEST Experiments

With the exception of APT imaging, CEST-based studies of AD to date have been conducted using a variety of different animal models of AD pathology. With one exception, these are all transgenic mouse models.

Two of these models develop only tau pathology: rTg4510 [120] and the PS19 line of the P301S transgenic mouse [121]. Both of these express genes found in patients with frontotemporal dementia with parkinsonism and are models of tauopathy which develop memory impairment, NFTs, and neurodegeneration but not amyloid pathology.

Several models are based on combinations of APP and PS1 mutations found in familial AD, which develop either dense or diffuse amyloid plaques, as well as early stage tau pathology. These include APP/PS1 mice which express one of each type of mutation [106,122]. The choice of APP and PS1 genes affects both disease severity and time of onset, typically developing amyloid lesions similar to those seen in AD by 7–8 months [106]. The 5xFAD model expresses three different APP genes and two different PS1 genes, and is particularly aggressive, developing amyloid lesions at only 2 months [123]. APP23 mice do not express any PS1 mutations, but have a seven-fold overexpression of the human full-length APP (APP751) and develop dense amyloid plaques by 6 months [124].

The Tau4RΔK transgenic mouse model is based on Tau4R mouse, expressing a fragment of a human tau gene, crossed with an APP/PS1 mouse. This model develops both amyloid plaques and NFTs [125].

Finally, there is a rat model of induced Aβ pathology where an intracerebroventricular (ICV) injection of toxic soluble Aβ1–42 results in impaired memory, increased levels of tau, and neurodegeneration [126].

### 5.4. What Does Endogenous CEST Reveal in AD?

#### 5.4.1. APT as a Potential Indicator of Protein Aggregates and pH Changes

As Aβ and tau proteinopathies are the hallmarks of AD, it is not surprising that attempts have been made to use APT contrast for estimating the protein load in the brains of patients affected by this disease. There are at least three studies applying APT imaging for studying patients with AD-related dementia [127,128,129] (Table 4). Animal studies have been conducted in the transgenic rTg4510 mouse model of tauopathy [130,131], and a rat model of Aβ pathology [132] (Table 4).

Based on patient studies, voxel-wise comparison of APT maps reveals higher signal in amnestic mild cognitive impairment (aMCI) [128], and in fully developed AD [129]. Regional assessment in aMCI exhibits higher APT in hippocampus, white and gray matter within the temporal and occipital lobes, white matter in the frontal lobe, pons, thalamus, and putamen [128]. The significant increase in APT occurs in areas where tau accumulation begins in three of the four recently described phenotypes of tau-spreading pattern: MTL-sparing, posterior, or lateral temporal, in which the early onset of tau accumulation begins in either the temporoparietal and frontal cortices, the occipital lobe, or within the left-temporal area, respectively [23]. Moreover, based on ROC curve analysis, APT signal in the occipital lobe, as well as in the gray matter of the temporal lobe can be used as a biomarker for diagnosing aMCI with sensitivity of 83–89% and specificity of 72–78% [128]. In fully developed AD dementia with noticeable gray matter atrophy APT is reported to be elevated in hippocampus as compared to cognitively normal patients [127,129]. Wang et al. also has observed negative correlation between hippocampal APT and MMSE in AD patients [127], while Oh et al. report a similar correlation in the anterior cingulate for the whole study population, including healthy, MCI, and AD patients [129].

The opposite findings come from animal studies, in which lower APT is observed in hippocampus and cortex at the stage of dense NFT deposition in tauopathy [130,131]. Such a decrease of APT is also observed in atrophic areas of hippocampus and cortex, independently of the model used [130,131,132]. The percentage change in APT negatively correlates with the severity of tau load [130], and in Aβ pathology APT and APT_SAFARI_ in the hippocampus negatively correlate with GFAP-positive astrocyte density (astrogliosis assessed by glial fibrillary acidic protein, GFAP, immunostaining), and positively with short-term memory changes [132]. Wang et al. also report that APT_SAFARI_ signal is greater, and therefore easier to measure accurately, than conventional APT.

The discrepancies between the animal and human studies might come from many factors. First, the APT measured with MTR_asym_ includes contributions from many physical features, like pH, the molecular size of the peptide backbones, the peptides mutual configuration and condensation, all of which are magnetic field dependent and may be differently affected by B_0_. As a result, this metric has low specificity and must be interpreted carefully. To directly compare results from different studies, the saturation schemes used should be similar. The saturation scheme used in the human AD studies cited above is one typically recommended for examination of tumors, in which cellular density is very high and the increased cytosol protein results in hyperintensity in APT imaging [74]. In AD studies the APT contrast increases most likely as a result of protein aggregation, in accordance with in vitro findings of Aβ aggregates [133], in which the increase in MTR at 3.5 ppm is due to the semi-solid MT component which arises during the formation of Aβ oligomers. Using this particular saturation scheme, APT can be used as a biomarker for the prodromal stage of AD. As it negatively correlates with MMSE, hippocampal APT can be considered for diagnosis and monitoring of disease progression.

In the animal studies, the pulse characteristics were only provided in one study, in which a lower saturation power has been applied with a longer duration [132]. In normal tissue, the APT signal is negative at saturations powers below 2 μT [134] which may obscure any increase due to AD pathology. Reduced APT only in regions with high-ranked tau pathology may indicate that the presence of NFTs is attenuating chemical exchange between amide protons and water protons. Relatively small Aβ and tau proteins can result in distinct CEST peaks, however, at the onset of plaque formation, protein unfolding and aggregation cause this resonance to widen, and a decrease in the pure CEST effect can be observed [133,135]. The comparison of APT with APT_SAFARI_ suggests that eliminating the effects of direct water saturation, macromolecular and membrane MT and its asymmetry, as well as B_0_ inhomogeneities increases the potential to differentiate AD proteinopathy [136]. The decrease in APT in the animal models of AD might also reflect decreased pH caused by acidosis [75]. The lower pH decreases APT due to a slower exchange rate of amide protons, the amount of which is not changed [115]. This may be the case, as a recent post mortem human study show that pH is significantly lower in the extracellular space of AD brains and CSF as compared to age-matched controls [136]. The same study reveals that the cerebral extracellular pH significantly decreases with age in humans, as well as in C57BL6 mice (post-mortem data). In another study it has been shown that normal ageing, as well as AD progression, decreases the intracellular pH [137]. This finding would imply changes in the APT values according to both ageing and AD progression, however the studies in healthy elderly patients do not present consistent results [128,138]. In light of these findings, it is impossible to interpret the data from pathological studies unambiguously.

#### 5.4.2. Creatine CEST (CrCEST) for More Precise Identification of Altered pH

Creatine (Cr) is one of the major metabolites involved in energy metabolism [139,140]. The ratio of phosphocreatine (PCr) to Cr in brain determines the effectiveness of the energy circuit [139]. A decrease in cerebral PCr indicates inhibition of mitochondrial function, and a gradual reduction of Cr concentration is observed in psychiatric disorders during neuronal tissue deterioration [140]. However, MRS-based studies often use brain Cr concentration to normalize quantities of other metabolites, as Cr is assumed to be constant or not detectably different from healthy brain, which is also assumed in AD studies [141,142,143,144].

**Figure 3:**
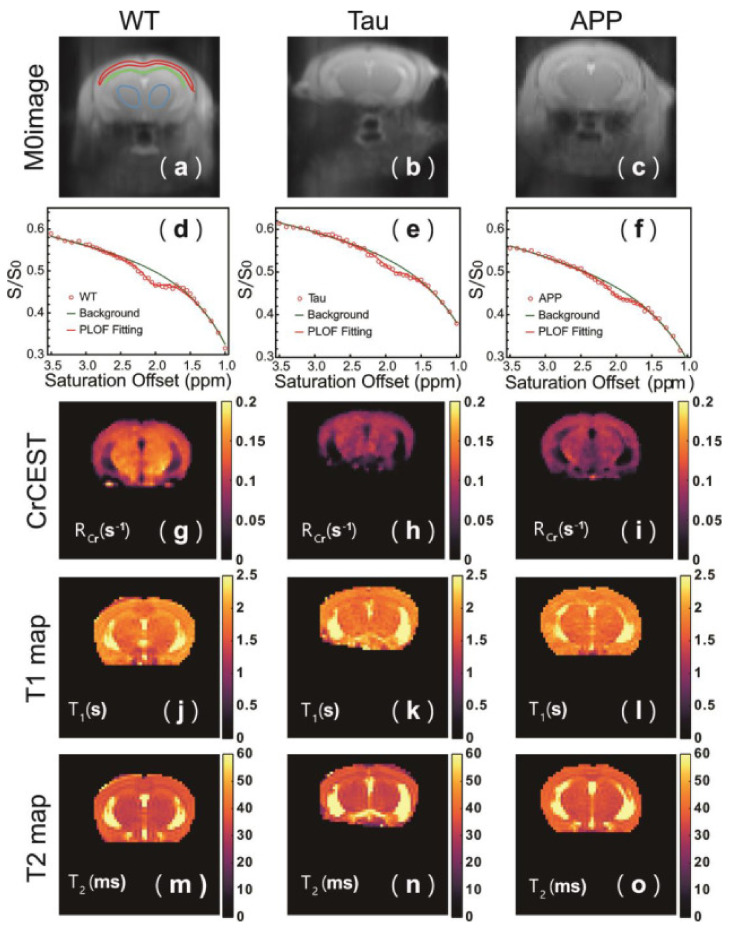
CrCEST imaging of mouse brain. Typical S_0_ images (**a**–**c**), cortical CrCEST Z-spectra (**d**–**f**), CrCEST maps (**g**–**i**), T_1_ maps (**j**–**l**), and T_2_ maps (**m**–**o**) for WT (**a**,**d**,**g**,**j**,**m**), Tau (**b**,**e**,**h**,**k**,**n**), and APP (**c**,**f**,**i**,**l**,**o**) mice. Both CrCEST Z-spectra and CrCEST maps (R_Cr_) of Tau and APP mice show a clear signal reduction compared to WT mice, while the T_1_ and T_2_ maps closely resemble each other among the three types of mice. The ROIs used to extract regional values are indicated in (**a**). The Z-spectra in d, e and f are from the cortical ROI in (**a**). [146] Reprinted from NeuroImage, 236, Chen et al., “Early detection of Alzheimer’s disease using creatine chemical exchange saturation transfer magnetic resonance imaging”, 118071, Copyright (2021), with permission from Elsevier, license no R5265S2SR6S5.

The CrCEST signal intensity depends not only on the metabolite’s concentration, but is also strongly related to pH [145], and so it cannot necessarily be used to quantify creatine. However, it may serve as a tool for investigating cerebral pH changes in AD. Chen et al. have investigated CrCEST in mouse models of AD: Aβ-related (APP/PS1 mice) and tau-related (Tau4RΔK mice) [146]. As a simple asymmetry analysis can confound results due to signals present on the opposite side of the spectrum, the authors use a different approach to analysis by converting their acquired z-spectrum into an R-spectrum: rotating-frame relaxation spectrum [147] to obtain the rotating frame relaxation rate (R_Cr_). In both models of AD, R_Cr_ is significantly decreased compared to wild type mice, and is associated with pathology in cerebral cortex, thalamus, and corpus callosum, but does not distinguish between the tau and Aβ-related models (Figure 3g–i). The CrCEST signal is similar between all groups. Quantification using both ^1^H MRS and ^31^P MRS also shows that Cr levels are similar between groups. The authors conclude that the R_Cr_ change in both AD models is due to a decrease in pH. Chen et al. further demonstrate that a reduction in mouse brain pH caused by hypercapnia results in a decrease in both CrCEST and APT, and use phantom studies to determine that both the CrCEST signal intensity and exchange rate, R_Cr_ at 2 ppm are linearly dependent on pH over a physiological range [146]. The region of R_Cr_ decrease observed in the APP/PS1 model corresponds to the areas where reactive forms of both astrocytes and microglia are present as confirmed by GFAP and IBA1 (Ionized calcium-Binding Adapter molecule 1) immunostaining. This suggests that the pH decrease originates from neuroinflammation preceding Aβ plaque formation. Interestingly, the Tau model showed no signs of neuroinflammation, but only a slightly elevated phosphorylated tau load, which suggests that other factors contribute to pH changes in early tauopathy.

Although just a single study (Table 5), Chen et al. [146] demonstrate that CrCEST contrast is a useful tool for detecting pH decreases in early stages of AD pathology in animal model of disease, and that these changes are associated with neuroinflammation in Aβ-related pathology. CrCEST is more sensitive to subtle changes in pH than APT contrast, since the exchange rate of guanidinium Cr protons is much greater than that of amide protons under physiological conditions [145], resulting in a more pronounced effect of pH on the CEST signal. It should be noted, however, that this method is valid for high magnetic field (7T and above), and that translating it to lower field clinical systems may be challenging because the in vivo CrCEST signal is then less separable from neighboring resonances [148].

#### 5.4.3. Neuroinflammation Detected by Myo-Inositol CEST (MICEST)

Brain myo-Inositol (mIns) has already been shown to reflect glial cell proliferation and activation [149]. Its presence in astrocytes is associated with osmoregulation, thus mIns elevation is observed in neuroinflammation [150]. Indeed, MRS studies of patients with different stages of AD reveal that elevated mIns/NAA and mIns/Cr are already present in the prodromal stage of AD, before CSF Aβ_42_ can be detected, and these parameters continue to increase as the disease progresses [142,143].

Myo-Inositol CEST (MICEST) studies have shown that in an APP/PS1 mouse model of advanced AD the MICEST contrast is twice that of healthy brain, which corresponds to ~50% increase in mIns/Cr assessed using MRS [151]. Importantly, this MICEST elevation is observed in the area of increased astrocyte activity, as confirmed by GFAP immunostaining [151]. Another study, in which an inflammatory challenge with lipopolysaccharide (LPS) has been performed in hippocampi of both APP/PS1 and WT mice, shows that MICEST increases in areas of glial activation, independently of the animal group [152]. The MICEST results from APP/PS1 mice are not distinguishable from WT perhaps because the study has been conducted in 3-month-old APP/PS1 mice, before the onset of Aβ overexpression. Moreover, it appears that MICEST depends linearly on the extent of microglia activation. The findings from the CEST studies by Harris et al. and Lopez et al. show the potential of MICEST to monitor glial cell proliferation with higher sensitivity than MRS, and with very good spatial resolution. This increases the usefulness of the method for mapping pathological changes [151,152]. Considering that early stage mIns changes occur in the APP/PS1 model and are clearly associated with astrocytic proliferation and activation, as demonstrated in another MRS-based study [141], the MICEST method brings the potential to detect early processes of astrogliosis in AD.

Some technical aspects should be taken into account while designing a MICEST study, especially while choosing the saturation parameters (Table 5, MICEST section). The mIns CEST effect at physiological pH is most pronounced with a B_1_ of 1–4 μT and saturation time of 1–4 ms [116]. Increasing saturation pulse amplitude, B_1_ causes a broader direct water saturation line, which overlaps with the MICEST peak and results in decreased efficiency of saturation transfer. This may cause underestimation of explicit mIns CEST effect and, consequently, underestimation of the metabolite’s pathological changes. Moreover, glucose and glycogen both have -OH groups which contribute to MICEST contrast [153,154]. The impact of glucose can be important, since the energy metabolism impairment in AD is reflected in changes of glucose consumption (please, see Section 5.4.1). Moreover, the abundance of tails from amine peaks should not be ignored. Specifically, the creatine and glutamate may be of interest since their contribution to the mIns resonance is substantial [116], and variations in these metabolites are also implicated in neuroinflammation, which was mentioned in the previous section (Section 5.4.2) concerning CrCEST, and will also be discussed in the next part of this review (gluCEST).

**Table 5:** Technical details of animal studies performed with the use of CrCEST, MICEST, and gluCEST imaging.

B_0_ (T)	Subjects ^1^ (AD/Early Stage AD/WT)	CEST Scans (Slices/Offsets), Offset Range	Saturation Power (μT)/Sat. Time (ms)	Parametrization	Brian Regions without Significant Differences	Brian Regions with Significant Differences	Ref.
**CrCEST**							
11.7	Tau4RΔK and APP/PS1 (0/7+7/5)	1/27, 2.3 to 5 ppm	2/1000	R_Cr_ ↓, ΔZ_Cr_ depend on pH, but N/S in AD	N/A	cortex, thalamus, corpus callosum	[146]
**MICEST**							
9.4	APP/PS1 (5/0/5)	1/20, 0 to 2 ppm	75 Hz/5000	MTR_asym_(0.6 ppm) ^2^↑	N/A	whole-brain, thalamus	[151]
9.4	APP/PS1 (0/6/6)	1/40, −4.00 to +4.00 ppm	0.9/1600	MTR_asym_(0.6 ppm)↑ in neuroinflammation and astrogliosis, correlates with density of reactive microglia	N/A	hippocampus	[152]
**gluCEST**							
9.4	APP/PS1 (6/0/6)	1/50, −5.00 to +5.00 ppm	250 Hz/1000	MTR_asym_(3.0 ppm)↓, correlates with MRS-derived Glu/tCr		hippocampus	[155]
9.4	PS19 (9/0/8)	1/40, ± (2.4 to 3.6) ppm	5.9/4 × 250	MTR_asym_(3.0 ppm)↓, correlates with synaptic density	cortex, hippocampus DG	hippocampus CA and thalamus	[156]
9.4	PS19 (6/6/9)	3/10, ± (2.5 to 3.5) ppm	5.87/4 × 250	early: MTR_asym_(3.0 ppm)↑; advanced: MTR_asym_(3.0 ppm)↓, correlates: (+) with synaptic density and (−) with density of reactive microglia		early: CA1 and DG subregions; advanced: all hippocampal layers	[157]
7.0	5xFAD (23/6/29)	2/50, −5.00 to +5.00 ppm	5/8 × 100	early & advanced: MTR_asym_(3.0 ppm)↓, correlates with synaptic and neurites density	caudate	early: parietal and temporal cortex, hippocampus; advanced: frontal cortex, thalamus	[158]

^1^ “Subjects” column lists the model used, and the numbers of subjects are given in brackets. ^2^ MTR_asym_ (0.6 ppm) defined as: $\frac{S\left(-0.6\mathrm{ppm}\right)-S\left(+0.6\mathrm{ppm}\right)}{S\left(+20\mathrm{ppm}\right)}\times 100\%$. CrCEST—creatine chemical exchange saturation transfer (CEST); MICEST—myo-Inositol CEST; gluCEST—glutamate CEST; R_Cr_—rotating frame relaxation rate for creatine CEST resonance; ΔZ_Cr_ = S_sat_/S_0_ for creatine CEST resonance (S_0_—signal without saturation; S_sat_—signal with saturation); DG—*dentate gyurs*; CA and CA1—*cornu ammonis* and its subregion 1; ↑: increase of the measured parameter; ↓: decrease of the measured parameter.

#### 5.4.4. Glutamate CEST (gluCEST)—The Most Comprehensive Tool for Staging AD

Glutamate is a primary excitatory neurotransmitter. The major involvement of this metabolite is synaptic transmission [159] and it affects cognitive abilities, learning, and memory. Many animal and human studies have shown that glutamate flux via synaptic connections is highly variable and the metabolite’s concentration decreases in AD [144,160]. Moreover, the Glx (Glx = Glu + Gln) level in the cingulate region correlates with MMSE scores and Instrumental Activities of Daily Living Scale (ADL) in patients with AD [161]. Investigating glutamate alterations with CEST has not yet been reported in AD patients, however recent studies in animal models of AD show the potential of this method.

The earliest-stage Aβ-related AD findings show that a decrease in gluCEST signal begins as early as one month of age in parietal and temporal cortex, and in hippocampus. The signal was continuing to decrease at 15-months in an aggressive Aβ-related mouse model (5xFAD) and also spreads to frontal cortex, striatum, and thalamus [158]. This GluCEST contrast correlates with neurite density in parietal cortex in mild stages of AD (7-month 5xFAD model) but this correlation does not achieve significance in hippocampus [158]. Additionally, in this mild phase of Aβ-related pathology, the gluCEST signal shows a correlation with synaptophysin density in parietal cortex and hippocampus, while no signs of atrophy are reported at this stage [158]. A hippocampal gluCEST decrease of ~31% in another mature Aβ-related mouse model, APP/PS1, is also reported by Haris et al. [155]. Positive correlation of gluCEST with Glu/Cr ratio measured by MRS confirms the accuracy of the gluCEST method for detecting glutamatergic system failure in this advanced stage of AD, in which neuronal loss and neuroinflammation coexist as expressed in decreased NAA and increased mIns concentrations revealed by MRS [155].

The study by Igarashi et al. also shows that the decrease in gluCEST correlates with reduced cerebral blood flow (CBF) in Aβ-related pathology based on the 5xFAD model, independently of the disease stage and brain area, implying that gluCEST contrast is decreased in cerebral tissue hypoperfusion [158].

**Figure 4:**
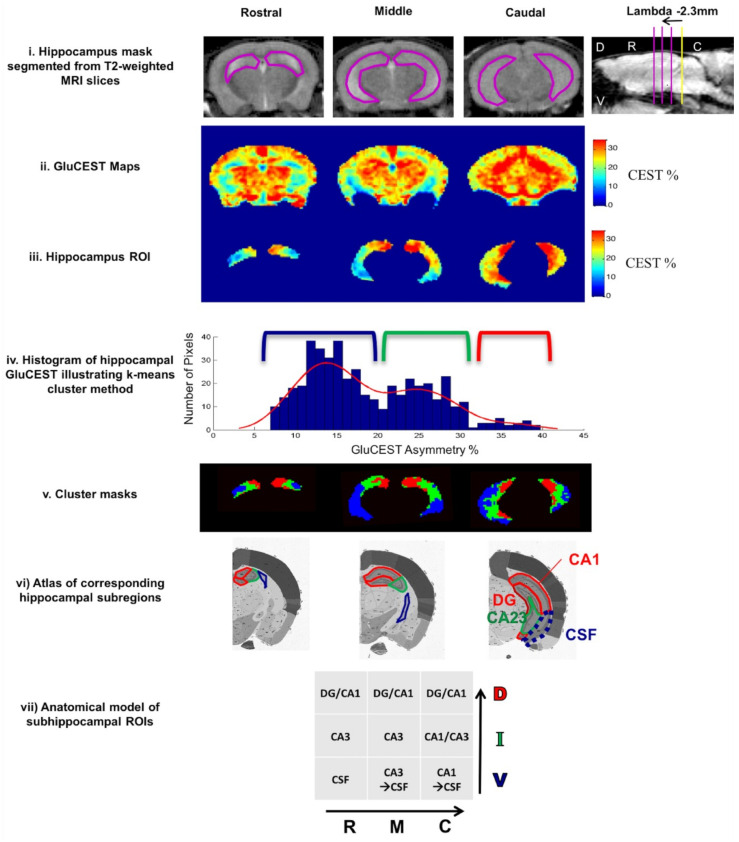
Image acquisition and segmentation pipeline for gluCEST imaging of mouse brain. (**i**) Anatomical T_2_-weighted magnetic resonance images (MRI) acquired from a live mouse brain. The example images shown here are from a 3 month old PS19 mouse. Slice positions were chosen relative to the lambda marker (yellow) identified on the sagittal scout image. The three slices (1-mm thick each) span the hippocampus from rostral to caudal. (**ii**) gluCEST asymmetry maps represent glutamate levels in vivo with high spatial resolution (156 μm^2^ inplane). Gray matter regions such as the thalamus have higher glutamate levels, compared to white matter regions like the corpus callosum where glutamate levels are low. (**iii**) Hippocampus proper segmented from the T_2_-weighted MRI and applied to gluCEST maps represents the hippocampus region of interest (ROI). (**iv**) Histogram of gluCEST values in an example slice of the hippocampus. The k-means clustering algorithm segments three “clusters” or subregions within the hippocampus based on high, medium, and low gluCEST values. (**v**) The three clusters are anatomically correlated to the dorsal (D, red), intermediate (I, green), and ventral (V, blue) hippocampus. The cluster mask displays the final subhippocampal ROIs. (**vi**) Three corresponding slices of the hippocampus from the Allen Brain Atlas [164]. Hippocampal subregions of interest are outlined: *dentate gyurs* (DG), *cornu ammonis* (CA1 and CA3), and the cerebral spinal fluid (CSF). Each region is colored to indicate their approximate cluster position. (**vii**) An approximate anatomical model of the subhippocampal ROIs which contribute to each cluster (dorsal, intermediate, ventral) in each slice (rostral, middle, caudal). In younger mice the CA1 extends through the caudal–ventral hippocampus, which later becomes CSF as mice age (arrow). © Crescenzi et al., 2017 [157], reproduced under permission of John Wiley and Sons, Inc. license no 5135830596118.

While applied to different stages of tauopathy in the PS19 mouse model, gluCEST contrast can be an accurate indicator of various altered processes in hippocampus during disease development [157]. In a very early stage of pathology elevated gluCEST is present in hippocampus and is associated with excitotoxicity [162]. This elevated gluCEST contrast is maintained throughout the disease progression in part of *cornu ammonis* (CA1) and *dentate gyrus* (DG) hippocampal subregions, as increased astrocytic activity arises to dispose of the excess of glutamate (confirmed by increased GFAP). Simultaneously, progressive loss of the gluCEST contrast is reported in caudal-ventral hippocampal regions (mainly CA1 and CA3 areas) [157]. This change reflects a decrease in glutamate concentration, also as a result of gliosis (increased GFAP), and points to reduced glutamate metabolism in synaptic mitochondria, as revealed by reduced synaptophysin concentration in these areas [157]. Similar findings are reported in another study of deeply developed tauopathy in a PS19 model, in which gluCEST in DG is unchanged [156]. The constant gluCEST signal in DG may imply that neurogenesis in DG compensates for the loss in gluCEST signal resulting from diminished synaptic integrity, so gluCEST is not sensitive to tauopathy in this region. On the other hand, gluCEST contrast seems to be independent of tau protein accumulation and is diminished in layers of synaptic deterioration before neuronal loss and long before tau pathology [156]. The above two studies by Crescenzi demonstrate that gluCEST is a suitable method for in vivo imaging of the glutamate gradient along the longitudinal axis of the hippocampus (Figure 4). It has to be noted, however, that PS19 transgenic mice serve also as a model of frontotemporal dementia [163], so further studies are needed to prove the utility of gluCEST in differential diagnoses of AD.

Also worth mentioning, age-dependent decrease in gluCEST contrast is also present in mature WT mouse hippocampus and parietal cortex, but to a lesser extent than in AD animals, and seems to be caused by senile decay [157,158].

The above studies of AD models clearly show that gluCEST can be used as an early indicator of regional glutamatergic synapse loss and decrease of axons and dendrite density preceding neurodegeneration. The early stage increase of gluCEST contrast reflects increased extracellular glutamate concentration in CNS, which is associated with activation of glial cells in early stages of neuroinflammation [162], thus gluCEST seems to be sensitive to inflammatory processes. Importantly, high spatial resolution of gluCEST imaging enables hippocampal layers to be distinguished, which is a valuable tool in assessing complex processes taking place in its subregions. Based on the studies discussed here, gluCEST can also be a predictive biomarker for hypoperfusion in mild AD as a consequence of earlier aberrant neuronal excitability and increased glucose consumption. De novo synthesis of glutamate requires glucose, thus while glucose metabolism is impaired, glutamate synthesis in AD is slowed [158]. This strong coupling between glutamatergic activity and cerebral glucose metabolism [165,166], suggests that glucose CEST (glucoCEST) has an application in AD, as a complementary examination for gluCEST, which will be discussed below.

### 5.5. Impaired Metabolism Revealed by Exogenous Glucose CEST (GlucoCEST)

Glucose is the main source of cerebral energy [165]. Many neuropathological incidents contribute to impaired glucose metabolism throughout neurodegeneration, not only in Alzheimer’s disease [167,168], but also in dementia with Lewy bodies [169], Huntington’s disease [170], frontotemporal dementia [171], as well as to a lesser extent in Parkinson’s disease [172,173]. Glucose consumption is strongly associated with synaptic function [174], with oxidative metabolism in neurons and aerobic glycolysis in astrocytes [166]. Hypometabolism is a crucial diagnostic target in dementias, reflecting abnormalities of inter- and intra-cellular signaling [165], synaptic loss [34], mitochondrial damage [175], deficiencies in neurotransmitter systems [139,176], and interregional metabolic route disruptions [28,177], which takes place before evident neuronal loss and cerebral atrophy [177]. Compromised energy metabolism as a hallmark of neurodegeneration allows for differential diagnoses based on which brain regions are affected by impaired glucose consumption [173].

Studies of glucoCEST in patients with Alzheimer’s disease are still missing, however they have been conducted in animal models focused on either Aβ-related pathology (APP23, AD-Aβ25–35, and APP/PS1) or tauopathy (rTg4510 or Tau4RΔK) [130,178,179,180,181] (Table 6).

The dynamic glucose-enhanced (DGE) MRI experimental setup consists of baseline brain imaging, and then injection of glucose contrast bolus, after which the glucose uptake is continuously recorded by glucoCEST imaging. Most studies use D-glucose either in intravenous injection of 50% *w*/*w* solution [180,181], or in intraperitoneal injection [130,179]. CEST-based imaging is performed continuously during contrast infusion and then imaging is continued for another 30–60 min to register glucose retention and clearance from the brain.

**Figure 5:**
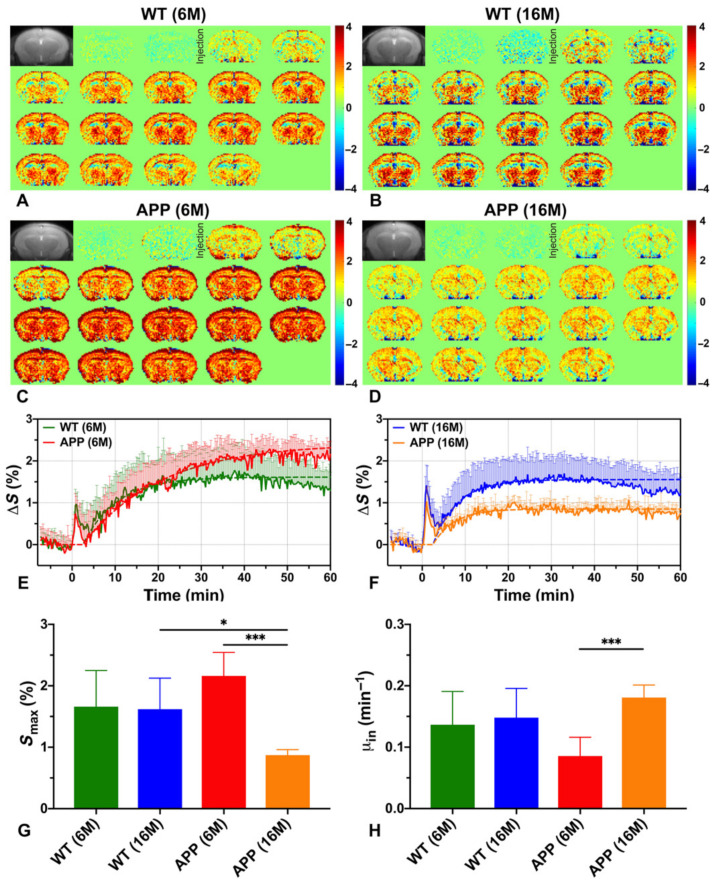
GlucoCEST imaging: Dynamic Glucose Enhancement (DGE) MRI results for brain parenchyma of WT and APP/PS1 mice. Dynamic difference images before and after D-glucose infusion for WT (**A**,**B**) and APP/PS1 (**C**,**D**) mice at 6 M (**A**,**C**) and 16 M (**B**,**D**). DGE images were averaged over sets of 15 for display (18 of 270). Experimental (solid line) and fitted (dashed line) parenchymal DGE curves for WT (6 M, *n* = 5) and APP/PS1 (6 M, *n* = 5) mice (**E**), as well as WT (16 M, *n* = 5) and APP/PS1 (16 M, *n* = 5) mice (**F**). Comparison of fitted uptake parameters *S*_max_ (**G**) and μ_in_ (**H**) between WT and APP/PS1 mice for two age groups (6 M and 16 M). Significance levels: * *p* < 0.05 and *** *p* < 0.001. Reprinted from [180]. © Huang et al., some rights reserved; exclusive licensee AAAS. Distributed under a CC BY-NC 4.0 license http://creativecommons.org/licenses/by-nc/4.0/ (access date 21 September 2021).

Based on these studies, the earliest signs of altered metabolism can be seen at the stage preceding Aβ plaques formation as a slower rate of D-glucose uptake in whole-brain parenchyma, thalamus, and entorhinal cortex in young AD animals (6-month, APP/PS1 model) as compared to age-matched normal control and older AD mice [180]. The whole-brain, and regionally cortical glucose consumption (S_max_), assessed as the maximum achieved within 60 min of injection, is higher in this AD phase versus control (Figure 5G). This is because of the DGE curve shape, which after reaching maximum maintains a plateau for 60 min in AD, while in control animals the curve starts to decrease after 40 min (Figure 5E). A similar glucose utilization curve is observed in older AD animals with severe Aβ protein aggregation in brain (Figure 5F), and is also reported by others, independently of Aβ or tau pathology [130,179,180]. Such dynamics indicate that glucose is not fully metabolized and accumulates in the tissue in both early and advanced stages of the disease. Wells et al. report that after 100 min of injection the cortical glucose uptake in developed tauopathy (9.5-month rTg4510 model) is higher than in healthy brain and is manifested in region of histologically dense NFTs and visible structural atrophy [130]. Interestingly, no glucose-utilization changes are present in areas with moderate tau pathology (such as hippocampus, even with atrophy). Cortical hypometabolism has been seen in a model of advanced tau-pathology (8-month Tau4RΔK) [181]. Advanced amyloidosis also results in lower glucose uptake in APP23, APP/P1, and AD-Aβ25–35 models in the cortex [178,179,180], as well as in the hippocampus, thalamus, and entorhinal cortex (APP23, APP/P1) [178,180], pointing to reduced glucose transport, which is in accordance to other studies [182]. Moreover, in the state of severe neurotoxicity in the AD-Aβ25–35 model, glucoCEST enhancement correlates with hippocampal concentration of mIns as measured by MRS, indicating that glucoCEST is also sensitive to inflammatory processes [179].

Interesting findings come from cerebrospinal fluid (CSF) glucoCEST imaging, which seem to be consistent independent of the AD model used. In young APP/P1 mice reduced clearance of D-glucose in CSF is reported as compared to young controls [180]. In mature APP/P1 animals with histologically confirmed severe Aβ protein aggregation [180], as well as in Tau4RΔK model with dense, diffuse NFTs [181] decreased CSF glucose uptake is observed relative to controls, with no visible washout within 30 min, in contrast to healthy brain.

Independently of the CEST technique used (standard continuous-wave or pulsed onVDMP) the available glucoCEST studies prove the method useful for detecting impaired glucose metabolism in AD models. Moreover, the utility of the on-resonance VDMP method for dynamic glucose enhancement measurements has been validated by MRS, showing comparable glucose uptake rate and level from these both methods [181]. Interestingly, the results are comparable while studying corresponding stage of pathology even in different models. The studies prove that naturally biodegradable glucose is a sufficient source of contrast in glucoCEST experiments. After injection, the blood glucose level reaches a maximum and then is stable within one hour. The brain glucose washout is not faster than the drop in blood glucose, allowing enough time for a comprehensive examination. The glucoCEST signal is higher in CSF than brain parenchyma and is similar to the glucose signal extracted from blood vessel ROIs. CSF-originating signal measured with the use of onVDMP imaging provides an opportunity for studying glymphatic system failure in AD, which is known to precede amyloid angiopathy and Aβ accumulation in cerebral parenchyma [183].

## 6. Conclusions and Future Remarks

While much work remains to be done, the results of ST-MRI studies are very encouraging, with potential to improve diagnosis and monitoring of AD (Table 7).

Magnetization transfer (MT) is sensitive to the changes caused by AD neuropathology, and may be helpful in differential diagnosis of AD, disease monitoring, and prognosis. Magnetization transfer ratio (MTR), a simple measure of MT, is consistently lower in AD subjects relative to healthy controls, particularly in the temporal lobe, hippocampus, and parahippocampus. Patterns of decreased MTR can help to distinguish AD from mild cognitive impairment or normal aging, as well as from other types of dementia such as dementia with Lewy bodies, vascular dementia, and non-AD medial temporal lobe atrophy. MT is well established and has already been implemented on most clinical systems. Improved analysis techniques, able to distinguish different patterns of MT changes, will be required in order for this to be useful in clinical practice. Quantitative MT (qMT)-based parametrization of brain tissue offers improved specificity of AD pathology even at the early stages of AD. However, qMT is much more complicated, time-consuming, and less well established than phenomenological MTR. For this reason, it will likely remain a research tool rather than standard clinical practice.

Several CEST MRI studies demonstrate that this modality is sensitive to some of the particular metabolic processes in AD. The increased specificity of CEST allows for studying several aspects of AD more selectively than MT, and at the very early stages of the disease. Specifically, gluCEST appears to be sensitive to neuroinflammation, CrCEST to pH changes associated with inflammatory status, while glucoCEST probes hypometabolism. GlucoCEST may therefore replace an FDG-PET examination, removing the radiation risk. All of this indicates that CEST methods have a great potential to assist in the development and monitoring of therapeutic treatments. The increased specificity of CEST for detecting particular molecular and metabolic alterations will likely also improve differential diagnosis of suspected neurodegenerative diseases.

CEST studies of AD to date also have some limitations. The majority of them use the conventional MTR asymmetry metric, which is characterized by low specificity as it contains contributions from many different molecular and relaxation characteristics of the tissue. Moreover, the utility of various CEST methods in patients still needs to be confirmed, as all but APT studies have been conducted only in animal models of AD and at a higher magnetic field than clinical scanners. Moreover, each of the animal models represent selective aspects of AD pathology and pattern of development that may differ from human disease. Thus, in order to better understand the complexity of AD pathology, it is necessary to develop animal models encompassing the widest spectrum of pathological processes and stages, including disease mediating factors. However, the non-invasive nature of most of the CEST studies means that human studies do not need to be delayed until the development of the perfect animal model of AD.

## Figures and Tables

**Table 1:** Human studies conducted using MT MRI.

B_0_ (T)	Subjects			Other	Regions	Ref.
	AD	MCI	NC			
1.5	23	0	16		WM ROI (1)	[83]
1.5	35	0	23	14 VaD & 13 other dementias	GM ROI (1)	[84]
1.5	38	0	21		GM ROI (1)	[85]
1.5	15	15	15		GM & WM (separately & combined): WB	[86]
1.5	18	0	16		GM only: WB, subregions (3)	[87]
1.5	25	13	28		GM & WM combined: WB, subregions (2)	[88]
1.5	31	0	18	17 DLB	ROIs (4)	[89]
1.5	55	19	43		GM & WM (separately & combined): WB	[90]
1.5	55	19	43		GM & WM separately; subregions (4)	[91]
1.5	18	0	18		GM & WM combined: WB, ROIs (3)	[92]
1.5	1	5	14	PS1 mutation carriers	GM only: WB, subregions (4)	[93]
1.5	36	0	19	longitudinal treatment with memantine	GM & WM combined: WB, subregions (4)	[94]
3	15	0	15		Superficial WM only: subregions (39)	[95]
1.5	20	27	30		voxel-wise WB, subregions (2)	[96]
3	0	42	42	MCI—single vs. multiple cognitive domains	voxel-wise: WB conjunction & disjunction analysis	[97]
3	77	0	77		GM & WM separately: WB, subregions (6)	[98]

B_0_—static magnetic field strength of the imaging system; AD—Alzheimer’s disease; MCI—mild cognitive impairment; NC—normal control, VaD—vascular dementia; DLB—dementia with Lewy bodies; PS1—presenilin 1 protein; GM—gray matter; WM—white matter; WB—whole brain; ROI—manual ROI placed inside a specific brain structure, subregion—VOI including an entire lobe or specific brain region.

**Table 2:** Studies of qMT in human brain. qMT parameters which were considered to be significantly different in AD subjects relative to controls are in bold.

B_0_ (T)	Subjects (AD/MCI/NC)	MT Scans (Powers/Offsets)	Parameters Fitted ^1^	Brain Regions without Significant Differences	Brain Regions with Significant Differences	Ref.
1.5	14/0/14	10 (3/6)	M_0_^A^, T_2_^B^, T_1_^A^/T_2_^A^↓, F × T_1_^A^↓	parietal WM	bilateral hippocampus	[100]
1.5	12/10/22	7 (1/7)	T_1_^A^↑, T_2_^A^↑, M_0_^A^, T_2_^B^↓, R × M_0_^B^↓, F↑	hippocampal head	hippocampal body	[101]
3	19/0/11	12 (6/5)	T_1_^A^, T_2_^B^, R × M_0_^B^↓, F,	voxel-wise analysis of the whole brain—remainder of the brain	hippocampus, thalamus, posterior cingulate, insula, posterior parietal and occipital cortices	[99]
1.5	18/18/18	7 (1/7)	T_1_^A^, T_2_^A^, T_2_^B^, R × M_0_^B^, F (classifier ^2^)	hippocampal head, insula	entorhinal cortex, hippocampal body, temporal cortex	[102]
3	43/34/21	12 (6/5)	T_1_^A^, R × M_0_^B^↓, F	right inferior longitudinal fasciculus, right superior cingulum, uncinated fasciculus	left inferior longitudinal fasciculus, left superior cingulum, bilateral inferior cingulum	[103]

^1^ Studies which reported forward and reverse exchange constants are listed here as R × M_0_B. ^2^ Weist et al. 2013 used a classifier algorithm which was most accurate when using all qMT parameters. MT—magnetization transfer; M_0_—spin density; T_1_—longitudinal relaxation time; T_2_—transverse relaxation time; all of these parameters are defined for: A—free water pool (liquid) and B—bound water pool (macromolecular); R—magnetization exchange rate between the two pools; F—the ratio of the macromolecular pool size to the water pool; ↑: increase of the measured parameter; ↓: decrease of the measured parameter.

**Table 3:** Studies of MT in various animal models of AD.

B_0_ (T)	WT	AD Model				Regions Investigated	Ref
		APP/PS1	Tg2576	Other			
7	8	7			12	Cortex, hippocampus, whole brain	[109]
9.4	5		4		12	Cortex, hippocampus	[108]
	4		5		4, 6, 10	Cortex, hippocampus	
	3		2	3 Tg/SOD	11–14	Cortex, hippocampus	
9.4	11	10			18	neo-cortex, retrosplenial cortex, hippocampus and thalamus	[110]
	9			10 BRI	18		
7	11	16			2	posterior cortex, caudate, putamen, genu, anterior cortex, hippocampus, thalamus, hypothalamus, amygdala, splenium	[111]
	10	16			4		
	11	16			6		
	19	19			8		
	10	9			24		

WT—Wild type non-transgenic littermates of the AD models; APP—amyloid-β precursor protein; PS1—presenilin 1 protein; SOD—superoxide dismutase 2; BRI—amyloid-BRI protein.

**Table 4:** Technical details of studies using APT imaging for studying both patients and animal models of AD. Patient studies have been conducted with similar acquisition setup and their results can be compared directly. The animal studies use different experimental and data analysis approaches, and thus it is difficult to discuss their results with respect to human studies.

B_0_ (T)	Subjects (AD/MCI/NC)	CEST Scans (Slices/Offsets), Offset Range	Saturation Power (μT)/Sat. Time (ms)	Parametrization	Brian Regions without Significant Differences	Brian Regions with Significant Differences	Ref.
**APT—human studies**							
3.0	20/0/20	1/3, −6.00 to +6.00 ppm	2/4 × 200	MTR_asym_ (3.5 ppm) ↑	temporal white matter (TWM), occipital white matter (OWM) and cerebral peduncles (CPs)	bilateral hippocampus	[127]
3.0	0/18/18	4/32, −6.00 to +6.00 ppm	2/800	MTR_asym_ (3.5 ppm) ↑	hippocampus, frontal lobe GM, entorhinal cortex, and caudate nucleus	hippocampus, WM & GM in temporal and occipital lobes, the pons, frontal lobe WM, thalamus, and putamen	[128]
3.0	19/9/13	Whole-brain 3D/38, −5.00 to +5.00 ppm	2/4 × 200	MTR_asym_ (3.5 ppm)↑, APT peak (3.5 ppm) from six-pool Lorentzian fitting	parahippocampal gyrus, pons, precuneus	anterior cingulate, hippocampus, putamen	[129]
**APT—animal studies ^1^**							
9.4	rTg4510 (10/0/9) ^1^	1/79, −6.00 to +6.00 ppm	Not provided	AUC MTR_asym_ (3.3–3.7 ppm)↓	thalamus	cortex, hippocampus	[130]
9.4	rTg4510 (20/0/10) ^1^	1/79, −6.00 to +6.00 ppm	Not provided	AUC MTR_asym_ (3.3–3.7 ppm)↓	thalamus	cortex, hippocampus	[131]
7.0	Rat ICV injection of Aβ (10/0/10) ^1^	1/32, −6.00 to +6.00 ppm	1.3/4000 for APT and APT_SAFARI_	MTR_asym_ (3.5 ppm)↓, MTR_SAFARI_ (3.5 ppm)↓	thalamus	whole-brain, cortex and hippocampus	[132]

^1^ In animal studies, the “Subjects” column lists the model used, and the numbers of subjects in brackets indicate: fully developed AD/young animals with early-stage AD/wild-type control. APT—amide proton transfer; MTR_asym_—magnetization transfer ratio asymmetry; MTR_SAFARI_—magnetization transfer ratio asymmetry derived from APT SAFARI imaging; AUC—area under the curve; ICV—intracerebroventricular; ↑: increase of the measured parameter; ↓: decrease of the measured parameter.

**Table 6:** Technical details of animal studies performed with the use of glucoCEST.

B_0_ (T)	Subjects ^1^ (AD/Early Stage AD/WT)	CEST Scans (Slices/Offsets), Offset Range	Saturation Power (μT)/Sat. Time (ms)	Parametrization	Brian Regions without Significant Differences	Brian Regions with Significant Differences	Ref.
9.4	rTg4510 (5/0/5)	1/79, −6.00 to +6.00 ppm,	Not provided	AUC MTR_asym_ (whole range): S_max_(DGE)↑	hippocampus, thalamus, and whole-brain	cortex	[130]
7.0	APP23 (7/0/7)	1/58, −20.00 to +20.00 ppm	1.5/4000	AUC MTR_asym_ (2.3–1 ppm) & ΔZ(1.2 ppm): S_max_(DGE)↓	ventricles, whole-brain	cortex, entorhinal cortex, hippocampus and thalamus	[178]
3.0	APP/PS1 (5/5/10)	onVDMP sequence 1/1	3.1/60 ms for brain and 900 ms for CSF	ΔS(t), early: S_max_(DGE)↑, μ_out_↓ in brain and CSF; advanced: S_max_(DGE)↓, μ_in_↓ in brain, μ_in_ and μ_out_↓ in CSF	cortex, hippocampus	early: entorinal cortex, thalamus, whole-brain and CSF; advanced: all brain regions and CSF	[180]
11.7	Tau4RΔK (4/0/3)	onVDMP sequence1/1	3.1/36 ms for brain and 100 ms for CSF	ΔS(t): S_max_(DGE)↓, μ_in_↓ in brain and in CSF		whole-brainCSF	[181]
7.0	Rat ICV injection of Aβ (6/0/6) ^5^	1/79, −3.00 to +3.00 ppm	1.5/5000	MTR_asym_(0.9 ppm): S_max_(DGE)↓	hippocampus	Parietal cortex, and whole-brain	[179]

^1^ “Subjects” column lists the model used, and the numbers of subjects are given in brackets. S_max_ (DGE)—maximal signal S_max_ derived from dynamic glucose enhancement (DGE) curve; AUC—area under the curve; MTR_asym_—magnetization transfer ratio asymmetry; CSF—cerebrospinal fluid; onVDMP—on-resonance variable delay multiple pulse sequence; μ_in_ and μ_out_,—glucose uptake and initial clearance rates, respectively; ICV—intracerebroventricular. **↑**: increase of the measured parameter; ↓: decrease of the measured parameter.

**Table 7:** Summary of the revised saturation transfer MRI techniques with their potential usefulness in diagnosis and management of the pathology, their major advantages, and limitations.

ST-MRI Method	Number of Studies (Human/Animal)	Usefulness	Advantages	Disadvantages/Limitations
MTR	16/4	Differential diagnosis and longitudinal monitoring	- Clinically available - Relatively simple analysis	- No specific information about the nature of the pathology - Interpretation can be difficult
qMT	5/0	Research tool for understanding the mechanisms driving changes in MT signal intensity	- Potentially more sensitive than MT	- Long scan times and complex data analysis
APT	3/3	Differential diagnosis and longitudinal monitoring	- Increasingly available on clinical scanners - Most advanced of current CEST methods - Sensitive to a number of specific pathological changes associated with AD	- Conflicting results from animal studies - Several options for data analysis which could lead to inconsistent or conflicting results
CrCEST	0/1	Mapping of pH changes associated with neuroinflammation and astrogliosis	- Sensitive to very early stages of AD	- Not clinically available - Requires 3T or higher - Complex data analysis
MICEST	0/2	Mapping of neuroinflammation and astrogliosis	- Sensitive to very early stages of AD - Particularly sensitive to astrogliosis	- Not clinically available - Requires 7T or higher - Very difficult to measure accurately, with many confounding factors
GluCEST	0/4	Detection and monitoring of glutamatergic system failure, and imaging of functional connectivity	- Sensitive to very early stages of AD - Sensitive to a number of specific pathological changes associated with AD	- Not clinically available - Requires 7T or higher - Very difficult to measure accurately, with many confounding factors
GlucoCEST	0/5	Potential replacement for FDG-PET with no radiation risk	- Sensitive to very early stages of AD - Sensitive to impaired energy metabolism and glymphatic system failure - Data analysis is relatively straightforward	- Not clinically available - Requires an infusion of D-glucose or one of its analogues
